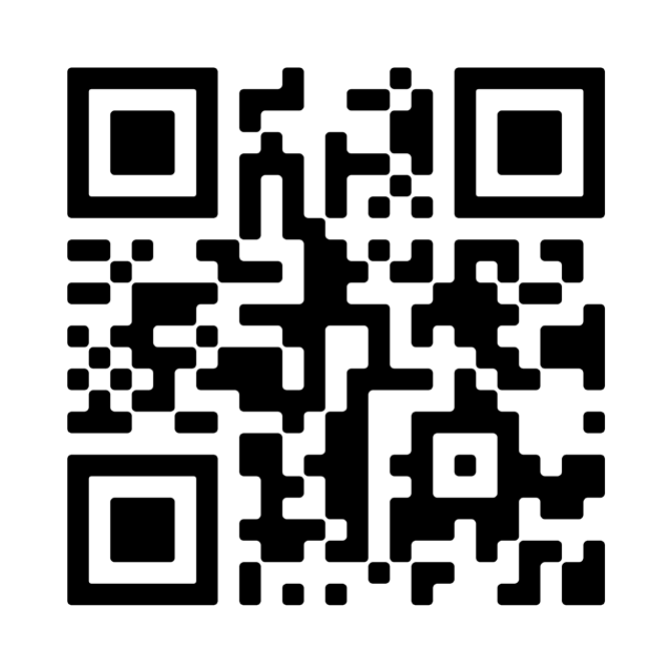# Surgical Treatment of Catamenial Chest Pain: Excision of diaphragmatic endometriosis during robot-assisted laparoscopic surgery

**DOI:** 10.52054/FVVO.14.4.048

**Published:** 2023-01-27

**Authors:** T Usta, M Gonenc, S Yilmaz, G.S.C. Akyol, A Kale, E Oral

**Affiliations:** Department of Obstetrics and Gynecology, Acibadem Altunizade Hospital, Acibadem University, Istanbul, Turkey; Department of General Surgery, Altunizade Hospital, Istanbul, Turkey - Department of General Surgery, Kent University, Istanbul; Department of Obstetrics and Gynecology, Acibadem Altunizade Hospital, Istanbul, Turkey; Department of Obstetrics and Gynecology, University of Health Sciences, Kartal Dr. Lutfi Kirdar Research and Training Hospital, Istanbul, Turkey; Department of Obstetrics and Gynecology, Bezmialem Vakif University, Istanbul, Turkey

**Keywords:** Diaphragmatic endometriosis, cyclical shoulder pain, robot-assisted laparoscopic surgery

## Abstract

**Background:**

10% of women of reproductive age are affected by endometriosis, and diaphragmatic endometriosis represents 1-1.5% of these cases. Diaphragmatic endometriotic lesions often require surgical treatment.

**Objectives:**

This video aims to demonstrate the appearance of diaphragmatic endometriosis and describe our experience with robot-assisted laparoscopic excision of full thickness diaphragmatic endometriosis.

**Materials and methods:**

The patient was a 37-year-old female with the complaint of cyclical right shoulder pain (for 1 year). She previously had caesarean section scar and umbilical endometriosis excision procedures. The magnetic resonance imaging (MRI) of the abdomen highlighted three endometriotic nodules, one of which was described as full thickness on the right hemi-diaphragm. The patient underwent a robot-assisted laparoscopic endometriosis surgery as a joint procedure between the gynaecology and general surgery teams. The falciform ligament was completely divided to obtain full views of the endometriotic lesions on the diaphragm. Superficial diaphragmatic lesions were first excised. The larger deep nodule, which was described on the MRI, was then excised with the full thickness of diaphragm. Pleural cavity was entered intentionally to achieve complete excision of the nodule. Laparoscopic assessment of the right lower pleural cavity through this opening did not show any endometriotic lesions. After the excision, the diaphragm was repaired with a barbed suture. Negative pressure suction of the pleural cavity was performed at the end of this repair instead of using a chest tube.

**Results:**

The patient was discharged on the 3rd day with no complications encountered. Histopathological examination confirmed endometriosis. The patient was asymptomatic three months after surgery.

**Conclusion:**

Robotic-assisted surgery is an easy and safe choice especially in such challenging dual compartment surgeries by providing a 3D view that abolishes sensory loss and increases depth perception, providing better manoeuvrability with tremor absence.

## Learning objective

This video aims to demonstrate the appearance of diaphragmatic endometriosis and describe our experience with robotic excision of full thickness diaphragmatic endometriosis.

## Introduction

10% of women of reproductive age are affected by endometriosis, and diaphragmatic endometriosis represents 1-1.5% of these cases. Even though it can be asymptomatic, patients may experience chest or shoulder pain, pneumo/haemothorax or haemoptysis (Nezhat et al., 2019). These symptoms are mostly catamenial (Selcuki et al., 2022). Diaphragmatic endometriotic lesions often require surgical treatment in symptomatic patients.

## Patients and Methods

The patient was a 37-year-old female with the complaint of cyclical right shoulder pain. There was no history of haemoptysis or cyclical chest pain. She previously had undergone excision of both post-caesarean scar and umbilical endometriosis. Physical examination was unremarkable. The magnetic resonance imaging (MRI) of her abdomen showed three endometriotic nodules, one of which was described as full thickness on the right hemi-diaphragm adjacent to liver. Computerised tomography (CT) of the thorax did not show any involvement of lung parenchyma. The patient underwent a robotic endometriosis surgery as a joint procedure by a gynaecologist and a general surgeon who had expertise in hepatic and diaphragmatic surgery. The procedure was performed in supine position. The patient was intubated with a double lumen tube to provide independent ventilation of each lung and produce an immobile surgical field. The first trocar of 8 mm was introduced in the umbilicus. Pneumoperitoneum with CO 2 was established with an intra-abdominal pressure of 14 mmHg. A 30-degree endoscope was used to increase visibility of the diaphragm in its entirety. Three more trocars of 8 mm were also introduced and connected to the robotic arms. An additional trocar was placed for assistance. A total of two endometriotic nodules, one on each hemi-diaphragm, were detected during the first exploration. The falciform ligament was completely divided in order to obtain a full view of the diaphragm. This dissection revealed another nodule which was infiltrating the right hemi- diaphragm. First, the nodule on the right diaphragm was resected. The branch of phrenic artery adjacent to the transmural nodule was sutured twice in order to prevent inadvertent bleeding. Full-thickness excision of the diaphragmatic nodule was performed. Pleural cavity was entered intentionally to achieve complete excision of the nodule. No implant was seen in pleural cavity during the inspection. The diaphragmatic defect was repaired with a delayed absorbable barbed suture. Decompression of the pneumothorax with negative pressure suction was performed at the end of the repair. The remaining superficial lesion of approximately 1 cm on the left hemi- diaphragm was excised carefully to avoid entering the pericardial cavity.

## Results

The operation was completed with no complication and the postoperative period was uneventful. The patient was discharged on the 3rd postoperative day with no complications encountered. Histopathology confirmed diaphragmatic endometriosis. The patient was asymptomatic at follow-up three months after surgery.

## Discussion

Even though it is uncommon and usually asymptomatic, diaphragmatic endometriosis can be incapacitating for some patients (Nezhat et al., 2019). Medical treatment is an option, however patients often need surgical management due to high failure rates and secondary side effects of medical treatment (Smith et al., 2021). Different surgical approaches such as laparotomy, laparoscopy, thoracotomy, thoracoscopy and robot-assisted laparoscopy have been described as treatment options. Laparoscopic surgery offers adequate visualisation of diaphragm and is superior to laparotomy for diaphragmatic endometriosis due to its advantages (Cela et al., 2014).

In a retrospective series of consecutive patients, Ceccaroni et al. (2013) concluded that laparoscopy is feasible and cost-effective for diaphragmatic endometriosis treatment (Cela et al., 2014). Robot-assisted surgery, on the other hand, besides providing greater precision and tremor absence, offers better excision and suturing capability with the instruments which can move with 540 degrees of angles in 7 different axes due to its endowrist technology. This technology allows the surgeon to perform the procedures more easily than conventional laparoscopy especially in narrow and deep locations.

Diaphragmatic endometriosis surgeries have a higher risk of cardiothoracic complications such as tension pneumothorax, cardiac contusion, or tamponade. In order to minimise these risks, a multidisciplinary approach is required. MRI is useful to diagnose and determine the extent of the disease preoperatively. This information is important for this multidisciplinary planning and preoperative preparation. Our patient had nodules which were adjacent to the liver and were infiltrating the diaphragm transmurally. We chose the robotic- assisted approach for our patient to facilitate surgery and minimise the risk of complications and injury to the adjacent organs by using the aforementioned advantages of this technique. In addition, a multidisciplinary approach to include a surgeon who is familiar with diaphragmatic surgery, intubation with a double lumen tube to ventilate each lung independently, use of a 30-degree scope and division of the falciform ligament were additional measures we undertook to perform this operation safely and effectively.

Some teams use video-assisted thoracoscopic surgery (VATS) in combination with laparoscopy for the management of diaphragmatic endometriosis. VATS allows direct visualisation of thoracic cavity and ability to resect the affected tissues. In our case, the patient had only shoulder pain. She did not have a history of haemoptysis or haemo/pneumothorax. On preoperative assessment, no lesion was detected on thoracic CT. Our examination through the diaphragmatic defect via the endoscope also did not reveal any endometriotic lesions. Therefore, we did not perform VATS.

We prefer direct closure of the defect after the excision whenever it is achievable, however in some cases with bigger defects, a mesh repair might be necessary. In our case we were able to close the defect with delayed-absorbable barbed suture which provided us with the convenience of suturing without the need for knots and keeping the continuous tension on the suture line. The resultant pneumothorax was decompressed trans- diaphragmatically and no chest tube was necessary after decompression (Bashir et al., 2010).

During our surgery, no complications occurred. The postoperative evolution was satisfactory.

## Conclusions

By providing a 3D view that increases depth perception and better manoeuvrability with tremor absence, robotic-assisted surgery appears to be an easier and safer choice especially in such challenging dual compartment surgeries. We presented our successful experience with robotic- assisted surgical treatment of diaphragmatic endometriosis. More cases and further studies are needed to demonstrate the appropriate role of this technique in diaphragmatic endometriosis.

## Video scan (read QR)

https://vimeo.com/780316397/8f4bd7556f

**Figure:**